# Towards pharmacokinetic boosting of phenoxymethylpenicillin (penicillin-V) using probenecid for the treatment of bacterial infections

**DOI:** 10.1038/s41598-024-67354-6

**Published:** 2024-07-21

**Authors:** Richard C. Wilson, Alaa Riezk, Paul Arkell, Damien Ming, Ryan Armiger, Victoria Latham, Mark J. Gilchrist, Anne-Grete Märtson, William W. Hope, Alison H. Holmes, Timothy M. Rawson

**Affiliations:** 1 National Institute for Health Research Health Protection Research Unit in Healthcare Associated Infections and Antimicrobial Resistance, Imperial College London, Hammersmith Campus, Du Cane Road, London, W12 0NN UK; 2 Centre for Antimicrobial Optimisation, Imperial College London, Hammersmith Hospital, Du Cane Road, Acton, London, W12 0NN UK; 3 Imperial College Healthcare NHS Trust, Hammersmith Hospital, Du Cane Road, London, W12 0HS UK; 4 David Price Evans Infectious Diseases and Global Health Group, The University of Liverpool, Liverpool, L7 8TX UK; 5 Leiden Academic Centre for Drug Research, Leiden University, 2333 AL Leiden, The Netherlands; 6 Antimicrobial Pharmacodynamics and Therapeutics Group, The University of Liverpool, Liverpool, L7 8TX UK

**Keywords:** Probenecid, Beta lactam antibiotics, Pharmacokinetics, Pharmacodynamics, Dose optimisation, Clinical microbiology, Bacterial infection

## Abstract

In the face of increasing antimicrobial tolerance and resistance there is a global obligation to optimise oral antimicrobial dosing strategies including narrow spectrum penicillins, such as penicillin-V. We conducted a randomised, crossover study in healthy volunteers to characterise the influence of probenecid on penicillin-V pharmacokinetics and estimate the pharmacodynamics against *Streptococcus pneumoniae*. Twenty participants took six doses of penicillin-V (250 mg, 500 mg or 750 mg four times daily) with and without probenecid. Total and free concentrations of penicillin-V and probenecid were measured at two timepoints. A pharmacokinetic model was developed, and the probability of target attainment (PTA) calculated. The mean difference (95% CI) between penicillin-V alone and in combination with probenecid for serum total and free penicillin-V concentrations was significantly different at both timepoints (total: 45 min 4.32 (3.20–5.32) mg/L *p* < *0.001*, 180 min 2.2 (1.58–3.25) mg/L *p* < *0.001*; free: 45 min 1.15 (0.88–1.42) mg/L *p* < *0.001*, 180 min 0.5 (0.35–0.76) mg/L *p* < *0.001*). There was no difference between the timepoints in probenecid concentrations. PTA analysis shows probenecid allows a fourfold increase in MIC cover. Addition of probenecid was safe and well tolerated. The data support further research into improved dosing structures for complex outpatient therapy and might also be used to address penicillin supply shortages.

## Introduction

The optimisation of antimicrobial dosing is of critical importance to ensure therapeutic success and minimise the harmful consequences of antimicrobial therapy, including toxicity and drug-resistant infections^1^. Delivery of optimal antimicrobial therapy requires an understanding of antimicrobial pharmacokinetics and pharmacodynamics (PK–PD). Achieving optimal drug exposure will support the desired clinical response in an individual with a susceptible bacterial infection.

Globally, there is a need to ensure that optimal antimicrobial PK are achieved using oral antimicrobials in the face of developing antimicrobial tolerance and resistance, which is observed as increasing minimum inhibitory concentrations (MICs)^2,3^. Current strategies for early oral antimicrobial therapy rely on highly bioavailable, broader spectrum agents, such as linezolid and fluoroquinolones^4,5^. These oral agents are associated with significantly higher rates of adverse events compared to narrow spectrum beta-lactams, such as penicillin, aminopenicillins, and first generation cephalosporins.

The optimisation of oral narrow spectrum antimicrobial dosing will support the core goals of the World Health Organisation (WHO) essential medicines list AWaRe criteria. The AWaRe criteria require narrow spectrum antimicrobials, such as penicillin, to be available in appropriate type, dose, and duration to treat common infections^6^. With increasing drug-resistance amongst common causative organisms, such as in streptococcal infections caused by Streptococci, new methods to optimise the delivery of “Access” agents and protect the use of broader “Watch” and “Reserve” antimicrobials are required^6,7^.

Probenecid, p-(di-*n*-propylsulfamyl)-benzoic acid, was developed in 1949 with the purpose of decreasing the renal clearance of penicillin^8^. The development of probenecid stemmed from the requirement to ration supplies of penicillin during World War II. Its mechanism of action is through competitive inhibition of organic anion transporters, which are responsible for excretion of organic agents, such as penicillin^9^.

Probenecid’s influence on penicillin clearance became mainly academic in the post-war era as our capability to produce more diverse, cheaper, and safer beta-lactam antibiotics rapidly expanded^8^. Probenecid remains a recommended adjunct to penicillin and cefoxitin in the management of sexually transmitted diseases such as neurosyphilis and gonococcal disease^10,11^. There is some evidence to demonstrate superior clinical outcomes and PK when added to oral beta-lactam antibiotics^12^. Current data on probenecid-boosted oral beta-lactam therapy is hampered because the analysis of beta-lactam PK was often determined based on total antimicrobial exposure from single drug doses and used old methods of quantification, such as tube dilution methods^12^. These methods were frequently open to wide variation and make direct comparison between studies challenging^12^. Furthermore, the use of total drug concentration does not allow for the active component (free drug concentration) to be described or understood, meaning that the true impact of probenecid on free antibiotic concentration remains to be defined in many cases^12^.

We undertook a randomised, open-label, cross-over study in healthy volunteers with the aim of describing both phenoxymethylpenicillin (penicillin-V) and probenecid PK in healthy volunteers, and the direct impact of probenecid on penicillin-V’s PK profile using gold standard analytical chemistry approaches. Using data from this study, we aimed to provide estimates of the potential improvement in penicillin-V PK-PD target attainment when boosted with probenecid.

## Methods

### Trial design and study setting

The study took place at Imperial College National Institute for Health Research (NIHR) Clinical Research Facility (CRF), Hammersmith Hospital, London, UK. We did a randomised, open-label, cross-over trial in healthy volunteers. Participants were randomised to receive either 250 mg, 500 mg, or 750 mg penicillin-V tablets four times a day in a ratio of 1:2:1 participants per dosing schedule. In clinical practice the normal dose of penicillin-V is 500 mg four times daily, but it can be given up to 1 g four times in severe infections^13^. We chose to test penicillin-V up to 750 mg four times daily with probenecid to safely allow an increase in maximal concentrations. The 1:2:1 ratio was to ensure the study was powered to test the most frequently used dose (500 mg four times daily)^14^. All participants received probenecid 500 mg tablets four times a day during their intervention visit based on previous studies deploying up to 2 g per day in divided doses^12^, and existing guidelines^11^. The order in which participants were allocated between the intervention (penicillin-V plus probenecid) and control (penicillin-V) arms was randomised. Participants were required to commence treatment 36-h prior to attending each study day (taking 5-doses pre-arrival at 0 h, 6 h, 12 h, 18 h, 24 h and 30 h). Between visits, a minimum of 7 days washout period was required. No changes to the protocol were made following commencement of the study. A copy of the study protocol is included in the Supplementary File. Results are reported in accordance with the CONSORT statement for randomised trials^15^.

### Ethical approval

This study was reviewed and approved by the Riverside Regional Ethics Committee (ref: 21/LO/0558) and the Medicines and Healthcare products Regulatory Agency (MHRA, EudraCT number: 2021-002800-11)**.** The study was registered on clinicaltrials.gov (registration number NCT05082909, registration date 19/10/2021). All the methods are in accordance with relevant guidelines and regulations.

### Participants

Participants were healthy volunteers recruited from the Imperial College NIHR CRF healthy volunteer database. An email advertisement was sent to the database, with respondents invited for screening. Informed consent was gained from all individuals screened. Participants were eligible for inclusion if they were adults over 18 years of age, had an estimated glomerular filtration rate (eGFR) > 90 mL/min/1.73 m^2^, and had ever previously taken a penicillin-based antibiotic. Participants were excluded if they had a known allergy to penicillin, other beta-lactam antibiotics, or probenecid, eGFR < 90 mL/min/1.73^2^, were pregnant, anaemic, abnormal liver function, lacked capacity, had a history of gout or uric acid kidney stones, G6PD deficiency, had evidence of an active infection, or took a regular medication that interacts with probenecid according to the SmPC.

At screening, baseline blood tests, biometric measurements (height and weight), and medical assessments were performed. Routine blood tests were not repeated at study visits unless there was a clinical indication (e.g. reported side effect) and was determined by the study principle investigator (TMR).

### Randomisation

Simple randomisation was performed using the randomizeR package (CRAN, version 3.0.1) in R. Randomisation was performed prior to commencement of recruitment by a member of the research team (TMR or RW). The generated randomisation list was kept on a central database available to all members of the research team. Upon recruitment and entry into the study participants were sequentially allocated to the randomisation list.

### Intervention

On study days, participants attended the CRF at midday. They took an observed (6th) dose of penicillin-V+/− probenecid depending on the visit. Time 0 was taken as the administration time of the observed dose. Phlebotomy was performed 45 min and 180 min post the observed dose. Blood samples were allowed to clot for 30 min. Clotted samples were spun a 3000 g for 5 min with serum immediately aliquoted and frozen at − 80 °C pending analysis. During the enrolment period, participants kept a paper dosing log and symptom diary, which was collected and reviewed at each visit.

### Penicillin-V and probenecid assays

Analysis was performed using high performance liquid chromatography/triple quadrupole mass spectrometry TQ LC/MS at Imperial College London Centre for Antimicrobial Optimisation using a published method^16^. Both total and free (unbound) drug concentrations were determined. Free penicillin-V and free probenecid concentrations were determined for individuals by performing ultra-filtration (Centrifree® ultrafiltration device, 30 kDa molecular weight cut-off, Sigma-Aldrich, Germany) prior to analysis. The limit of quantification for the TQ LC/MS assay was 0.01 mg/L for both penicillin-V and probenecid. The inter-day and intra-run performance of the assay met standards set out by the FDA and EMA^17,18^.

### Outcomes

The primary outcome for the study was total and free penicillin-V concentration as 45- and 180-min post an observed dose compared to probenecid-boosted penicillin-V. Secondary outcomes included (1) the development of a linked PK model describing the influence of probenecid on penicillin-V; (2) estimation of the probability of target attainment (PTA) for patients treated with penicillin versus probenecid boosted penicillin-V in the context of streptococcal infections; and (3) reported adverse events associated with control and intervention visits.

### Statistical methods

Data were recorded using OpenClinica, a fully Good Clinical Practice compliant electronic Case Report Form. Normality of data were assessed visually and using the Shapiro–Wilk test and described parametrically using a paired t-test and non-parametrically using the Wilcoxon signed-rank tests when appropriate. Proportions were compared using the Chi-squared test.

Non-compartmental and population pharmacokinetic analysis was performed using a Non-Parametric Adaptive Grid (NPAG) via Pmetrics in R^19^.

For population modelling of penicillin-V, PK data were combined with rich PK data for healthy volunteers receiving penicillin-V alone as previously described^20^. Two-, and three-compartment models with an additional absorption storage compartment were evaluated to describe total and free penicillin-V concentrations. First order and saturable absorption as well as first order and Michaelis–Menten clearance (Cl) from the central compartment were evaluated. Pharmacokinetic data were weighted by the inverse of penicillin inter-assay variance at the given concentration.

Age, height, weight (using actual body weight [ABW] or ideal body weight [IBW]), body mass index (BMI), creatinine (Cr), and creatinine clearance (CrCl; estimated using the Cockcroft-Gault equation separately for ABW and IBW), probenecid administration, and probenecid concentrations were evaluated as covariates for inclusion in the final model. Backward stepwise linear regression against individual PK parameters was used to explore correlations. Covariates were then evaluated using linear, exponential, power, and allometric models, where appropriate.

The fit of models to data were evaluated using (1) goodness of fit, (2) the coefficients of determination (r^2^), the y-intercept, and slope of regression from observed—versus predicted plots before and after the Bayesian plot; (3) the log-likelihood value; and (4) Akaike information criteria (AIC).

Target attainment within this study was assessed by simulating PTA for a range of dosing schedules. PTA defines the probability of a specific dosing schedule achieving a defined PK–PD target within the simulated population for a range of clinically relevant target organism minimum-inhibitory-concentrations (MICs)^21^. To estimate penicillin-V PTA for both penicillin-V only and penicillin-V plus probenecid Monte Carlo simulations (*n* = 1000 participants per dosing schedule) were performed using Pmetrics to determine the PTA with varying MIC for a range of doses. Dosing schedules simulated were 250 mg 6-hourly, 500 mg 6-hourly, 750 mg 6-hourly, 250 mg plus 500 mg probenecid 6-hourly, 500 mg plus 500 mg probenecid 6-hourly, 750 mg plus 500 mg probenecid 6-hourly. We evaluated this for a PK–PD target of 40%*f*T > MIC (proportion of the dosing interval which the free concentration of drug remains above the MIC) based on EUCAST rationale documents to achieve a two log drop in *Streptococcus pneumoniae*^22^.

### Adverse events

Adverse events were graded according to the Common Terminology Criteria for Adverse Events^23^.

### Sample size, interim analysis, and stopping rules

Using sparse PK sampling in a cross-over design previous estimates have suggested that a sample size of at least 13 participants is required to detect a binary covariate (i.e. 30% proportional increase in elimination clearance) with at least 80% statistical power and a significance of *p* = *0.05*. Prior studies, using different methodological approaches and penicillin-based antimicrobials have suggested that probenecid has approximately 30–40% effect on drug clearance^24^. Therefore, we planned to recruit up to a maximum of 50 participants to this study (providing 13 participants on each of 250 mg and 750 mg and 24 participants on 500 mg penicillin-V).

To ensure participant safety and that healthy volunteers were not exposed to additional risk of harm (either due to high rates of side effects or continuing unnecessary prolongation of the study once a highly significant difference had been observed) safety monitoring was undertaken by members of the research team at each study visit. Additionally, an a priori decision was made to perform interim data analysis of drug concentration data after every 10 participants had completed their two study visits. Based on interim data an updated power calculation would be performed with a plan to stop the study if the primary outcome results demonstrated a significance of *p* < *0.01* and power of > 99% for the current sample size.

## Role of funders in research

This report is independent research funded by the BIA and Department of Health and Social Care. The views expressed in this publication are those of the author(s) and not necessarily those of the Department of Health and Social Care, NHS, or the National Institute for Health Research.

## Patient consent

Ethical approval was granted by the Riverside Research Ethics Committee (21/LO/0558). Prior informed written consent was provided by all study participants before entry into the study.

## Results

### Study summary

**Figure 1:**
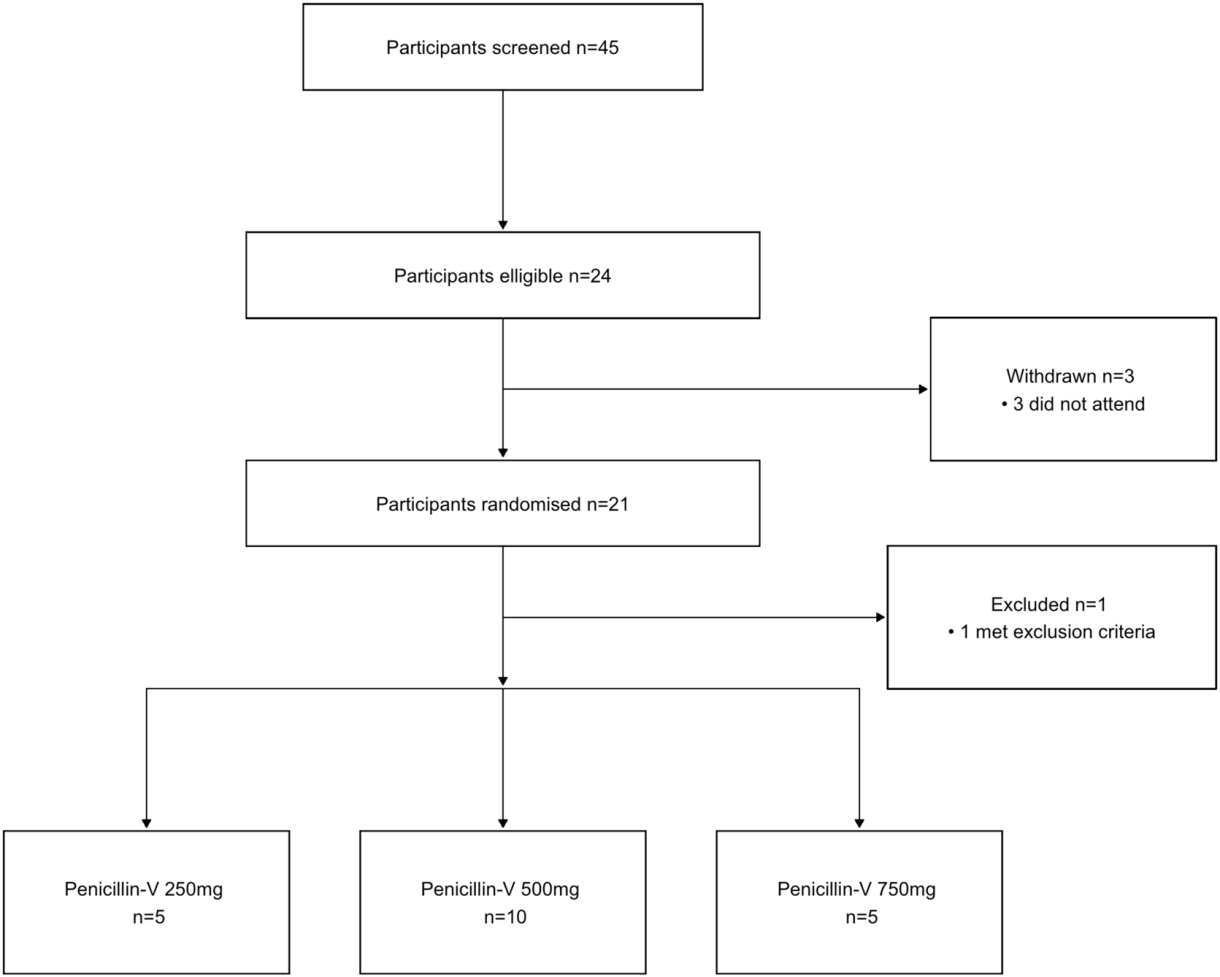
CONSORT diagram.

Between December 2021 and June 2022, 45 participants were screened for inclusion. In total, 21 participants were enrolled and randomised with one participant withdrawn from the study prior to their first dosing visit after meeting an exclusion criterion. Figure 1 summarises participant recruitment and flow. The study was closed in July 2022 following interim data analysis which demonstrated that our pre-specified stopping criteria were met after 20 participants had completed study visits.

### Baseline data

Baseline data for participants is summarised in Table 1. Participants’ mean (SD) age was 53 (17) years and 11 (55%) were male. Mean (SD) height was 171.5 (7.8) cm, weight 72 (9) kg, creatinine 68 (7) µmol/L, and albumin 41 (2) g/L. Participants were randomised to 250 mg (5/20, 25%), 500 mg (10/20, 50%), and 750 mg (5/20, 25%) doses of penicillin-V. All participants provided dosing diary cards. One (5%) participant missed their first dose of penicillin-V prior to both study visits. All participants had serum samples collected at 45-min and 180-min post their observed dose on both visits.

**Table 1:** Demographics.

		Total (n = 20)	250 mg (n = 5)	500 mg (n = 10)	750 mg (n = 5)
Age (years)	Mean (SD)	53 (17)	49 (22)	61 (10)	43 (17)
Sex	Male	11 (55)	2 (40)	5 (50)	4 (80)
	Female	9 (45)	3 (60)	5 (50)	1 (10)
Height (cm)	Mean (SD)	171.5 (7.8)	168.4 (9.4)	172.2 (7.9)	173.2 (6.8)
Weight (kg)	Mean (SD)	72 (9)	69 (4)	70 (8)	79 (12)
Creatinine (μmol/L)	Mean (SD)	68 (7)	63 (5)	69 (7)	72 (6)
eGFR (mL/min/1.73 m^2^)	Median (IQR)	98 (94–115)	116 (98–118)	94 (94–98)	113 (98–118)
Albumin (g/L)	Mean (SD)	41 (2)	41 (3)	41 (2)	42 (2)

*eGFR* estimated glomerular filtration rate (CKD-EPI).

### Primary outcome

**Figure 2:**
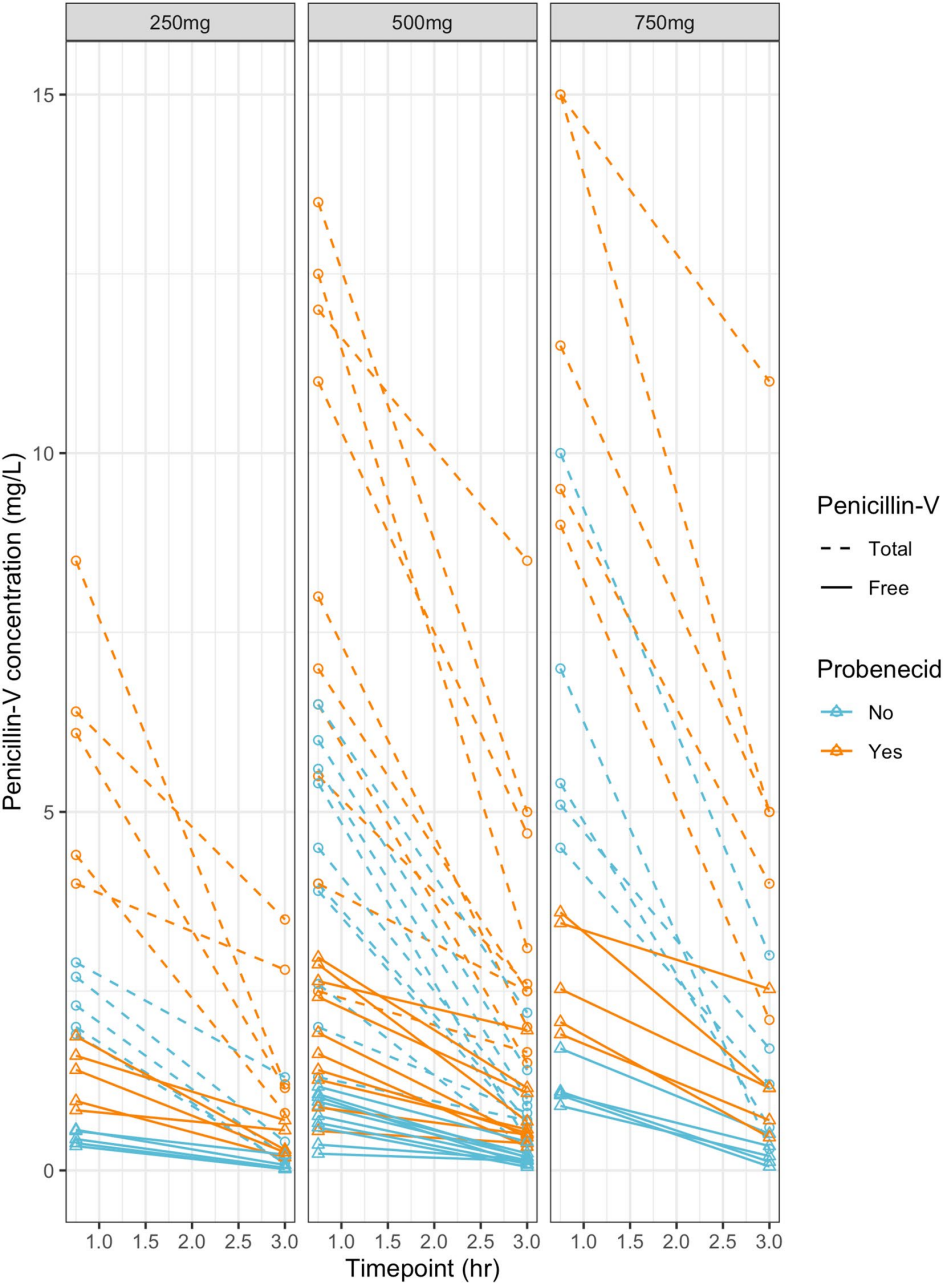
Spaghetti plot of penicillin-V concentrations, by dose.

Primary outcome data is summarised for all 20 participants in Tables 2 and 3. Serum total and free penicillin-V concentration was different at both 45- and 180-min between control and intervention arms (significant when tested in the 500 mg group, not significance-tested in the 250 mg and 750 mg due to low numbers). At 45-min, the mean difference (95% CI, *p* value) in total penicillin-V concentration between control and intervention arms was 4.32 (3.20–5.43, *p* < *0.001*) mg/L and the mean difference in free penicillin-V was 1.15 (0.88–1.42, *p* < *0.001*) mg/L. At 180-min the mean difference in total penicillin-V was 2.2 (1.58–3.25, *p* < *0.001*) mg/L and in free penicillin-V difference was 0.5 (0.35–0.76, *p* < *0.001*) mg/L. The mean (SD) free fraction of penicillin-V in this study was observed to be 18.6 (1.7) %. Penicillin-V concentrations are presented in a spaghetti plot in Fig. 2.

**Table 2:** Penicillin-V concentrations when administered alone and with probenecid.

		Total concentration (mg/L)			Free concentration (mg/L)		
Time	Dose*	Penicillin-V	Penicillin-V and probenecid	*p* value^†^	Penicillin-V	Penicillin-V and probenecid	*p* value^†^
45 min	250 mg	2.3 (2.0–2.7)	6.1 (4.4–6.4)	NA	0.43 (0.38–0.66)	1.40 (0.97–1.60)	NA
	500 mg	4.3 (2.9–5.6)	7.5 (5.8–11.8)	0.006	0.83 (0.62–1.01)	1.78 (1.30–2.60)	0.006
	750 mg	5.4 (5.1–7.0)	11.5 (9.5–15.0)	NA	1.07 (1.05–1.10)	2.53 (2.07–3.45)	NA
3 h	250 mg	0.20 (0.10–0.40)	1.20 (1.15–2.80)	NA	0.04 (0.02–0.08)	0.29 (0.25–0.56)	NA
	500 mg	0.75 (0.63–0.98)	2.55 (2.13–4.30)	0.006	0.17 (0.10–0.19)	0.55 (0.47–0.98)	0.006
	750 mg	1.20 (0.60–1.70)	5.00 (4.00–5.00)	NA	0.20 (0.12–0.34)	1.15 (0.70–1.15)	NA

Concentrations are median (IQR); *250 mg n = 5, 500 mg n = 10, 750 mg n = 5; ^†^Wilcoxon signed-rank test (not conducted where n ≤ 5).

**Table 3:** Probenecid concentrations.

		45 min	3 h	*p* value*
Probenecid concentration (mg/L)	Total	79.0 (22.0)	81.1 (21.1)	0.760
	Free	6.6 (1.5)	6.6 (1.6)	0.943

Concentrations are mean (standard deviation), *two sample t-test.

Mean (SD) free fraction of probenecid was 8.4 (1.0) %. There was no observed difference in probenecid concentration (total or free) at 45- and 180-min (total: 45 min 79.0 mg/L, 180 min 81.1 mg/L and free: 45 min 6.6 mg/L, 180 min 6.6 mg/L). The free fraction of probenecid also remained stable between 45- and 180-min timepoints (8.5% versus 8.3%, respectively).

### Secondary outcome

Three and four compartment models were evaluated with and without absorptive time lag. A first order error polynomial best fit our local assay variance and was combined with an additive model (lambda) to reflect other process noise. Observations were then weighted by 1/error^2^.

The time course of total concentrations of penicillin-V are best described by a three-compartment model (Eqs. 1–3 describe absorption, total, and peripheral compartments) shown schematically in Supplementary File 1A. Ka is an absorption rate constant describing total drug transfer into a central compartment. Penicillin-V has a total volume, V, and is cleared from the central compartment by a secondary variable, K_e_ = Cl1/V in the absence of probenecid and K_e_ = Cl2/V in the presence of probenecid. Probenecid was included as a binary covariate on clearance. A peripheral compartment describes transfer from the total compartment with coefficients K23 and K32. Compartments were zeroed between study visits assuming complete elimination over a seven-day drug-free period.

1 $$ \frac{dX\left( 1 \right)}{{dt}} = - Ka*X\left( 1 \right) $$

2 $$ \frac{dX\left( 2 \right)}{{dt}} = Ka*X\left( 1 \right) - \left( {Ke + K23} \right)*X\left( 2 \right) + K32*X\left( 3 \right) $$

3 $$ \frac{dX\left( 3 \right)}{{dt}} = K23*X\left( 2 \right) - K32*X\left( 3 \right) $$

Output equation

$$ Y\left( 1 \right) = \frac{X\left( 2 \right)}{V} $$

Parameter estimates, credibility intervals and shrinkage are shown in Supplementary File 1B. Observed versus predicted and goodness of fit plots are shown in Supplementary File 1C and 1D. The visual predictive check revealed good performance of the final model without significant deviations or outliers. Covariate analysis did not reveal any PK parameters which resulted in an improvement in fit and no other covariates were retained in the final model.

### Probability of target attainment

**Figure 3:**
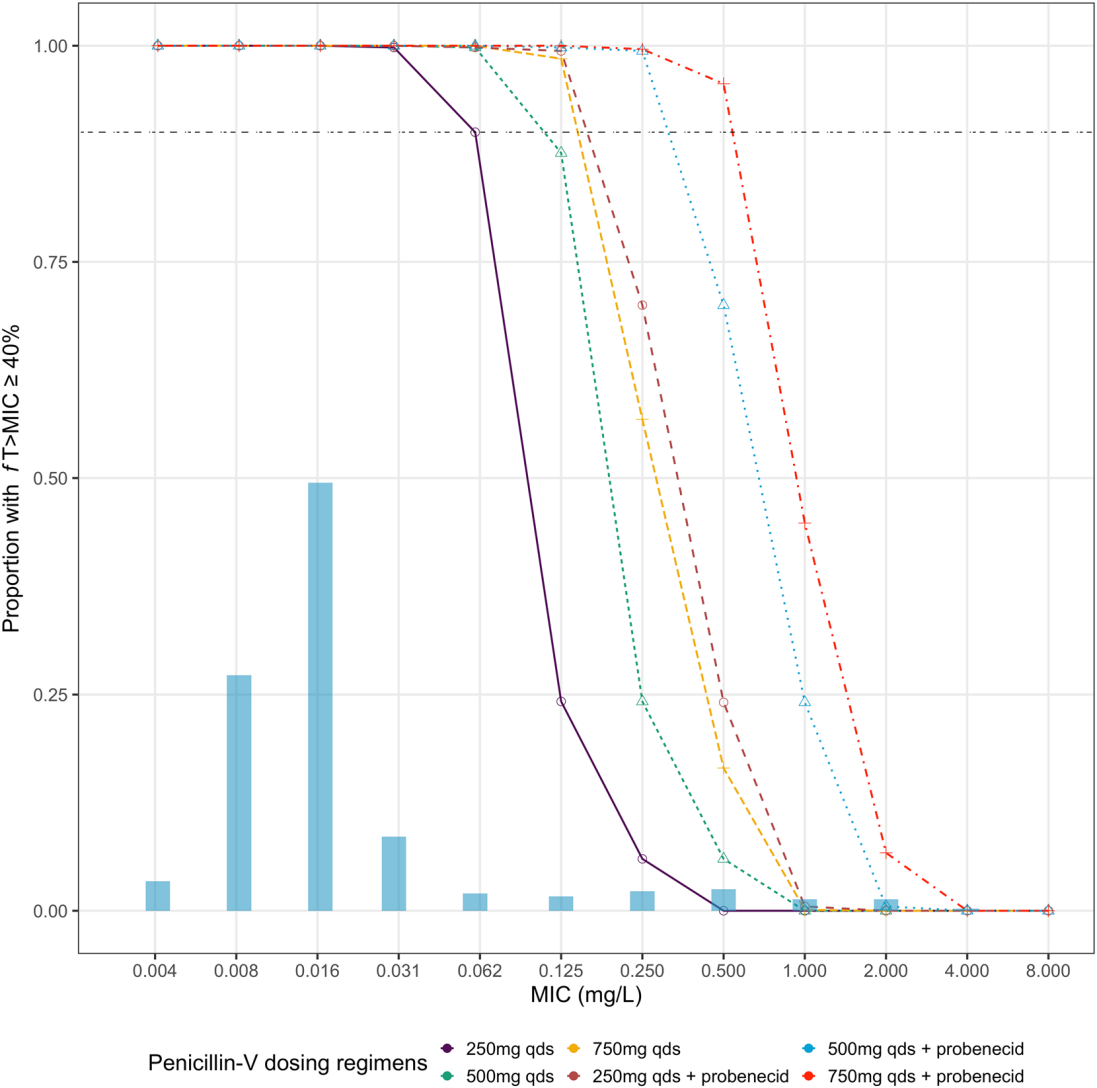
Probability of target attainment (40%*f*T > MIC) with overlaid MIC distribution of *Streptococcus pneumoniae* from EUCAST. The horizontal dashed black line shows where 90% of the simulated population meets the PKPD target. The blue horizontal bars show the EUCAST MIC distribution for S. pneumoniae. *f*T > MIC, proportion of the dosing interval which the free concentration of drug remains above the MIC; MIC, minimum inhibitory concentration.

Monte Carlo simulations (*n* = 1000 for each participant) were performed from the final PK model and PTA calculated based on 40%*f*T > MIC over a selection of relevant MICs. The free penicillin-V concentration was calculated from the mean population free fraction. Results are shown in Fig. 3. The EUCAST distribution for *S. pneumoniae* is overlaid in columns to provide context (https://mic.eucast.org/). The simulations generally suggest the addition of probenecid achieves an extra two dilutions equivalent to covering a four-fold increase in MIC.

### Adverse events

Twenty ADRs were reported in the study (19/20 mild, 1/20 moderate). One moderate ADR was reported in the probenecid group relating to a swallowing difficulty with the tablets. There were eight mild ADRs during the penicillin only group (3/8 nausea, 1/8 dizziness, 1/8 difficulty swallowing, 1 flu-like illness, 1 leg pain, 1 flushing) and 11 mild ADRs in the penicillin and probenecid group (4/11 nausea, 3/11 headache, 1/8 diarrhoea, 1 difficulty swallowing). No significant differences were found overall between groups.

## Discussion

This study found that the addition of probenecid to penicillin-V led to a significant increase in observed penicillin-V concentration and PTA for higher MIC organisms. We present both total and free concentration data for each drug, and a pharmacokinetic model describing the interaction. Probenecid was well tolerated with similar adverse events compared to penicillin-V alone.

These data are in line with recent reports by Everts and colleagues who demonstrated a significant increase in PTA against *Staphylococcus aureus* when flucloxacillin or cefalexin were administered alongside probenecid^25,26^.

Whilst the addition of probenecid to penicillin clearly increases drug-exposure, the pharmacodynamic and clinical implications of this observation warrant further investigation. There is much research into shorter versus long courses of antibacterials^27^ however the impact of individual patient dosing and drug-exposure is often less clear. Studies have shown non-inferiority for higher doses and shorter time periods for penicillin-V in Group A streptococcal pharyngotonsillitis^28^, levofloxacin in atypical community acquired pneumonia (CAP)^29^, and amoxicillin for children with CAP^30^. In these studies, PK analysis was not undertaken, meaning the mechanistic role of an exposure–response relationship cannot be assessed individually. Future studies will look to confirm whether probenecid could have a similar effect on shortening treatment duration and clearly defining the impact of exposure–response on this variable. Probenecid may also facilitate earlier intravenous to oral switch in cases where a higher drug exposure is required but unobtainable due to incomplete absorption or dose-limiting toxicity.

There are no clinical breakpoints for penicillin-V for the targeted treatment of *S. pneumonia*e. The only available breakpoints are based on penicillin-G (benzylpenicillin) susceptibility where an MIC > 0.06 mg/L but < 2 mg/L for non-meningitic indications requires increased exposure of penicillin-G, and > 2 mg/L implies penicillin-G resistance^31^. Our data provide reassurance that penicillin-V 500 mg four times daily adequately covers susceptible isolates of *S. pneumoniae*. With the addition of probenecid and doses of penicillin-V 750 mg four times daily our data suggest it would be possible to treat an isolate with a penicillin-V MIC up to 0.5 mg/L. Accordingly, in clinical settings with high rates of penicillin resistance the doses tested in this study are not likely to be appropriate for the empiric treatment of *S. pneumoniae*. However, the combination offers a therapeutic option for the targeted treatment of certain isolates within the susceptible, increased exposure range for penicillin.

Our data show ample redundancy in the PTA for penicillin-V against *S. pneumoniae* over the dose regimens studied. However, given the considerable increase in PTA at higher MICs the other application for further research in a clinical study is whether penicillin-V and probenecid in a reduced frequency regimen (e.g. twice daily) would be an acceptable alternative to four times daily currently used in clinical practice.

The optimal dose of probenecid has not been defined. Total and free probenecid concentrations were stable between the two timepoints within our study. This could be due to ongoing absorption of probenecid which has a time to maximal absorption of 1 to 5 h^32^. This combined with the limited time between sampling does not allow reliable estimation of half-life, but it has previously been shown to be dose dependent between 4 and 12 h^32^. Further accumulation of probenecid is therefore possible with longer courses and might have additional impact on penicillin-V clearance. The relevance of probenecid concentrations is to be explored and optimal dosing strategies to support probenecid will be evaluated in future work.

Consistent with previous studies^12^ penicillin-V and probenecid was well tolerated with mild nausea being the most frequently reported side effect. We encountered two instances of swallowing difficulty with probenecid which might have been due to the large tablet formulation used within this study.

Probenecid-boosted oral therapy may also have a role in the face of current penicillin supply shortages, such as the serious shortage protocols enacted by the UK government in response to rapidly increased penicillin consumption following the 2022–2023 Group A Streptococcus outbreak^33,34^. Probenecid is unlicensed in the UK and not routinely available outside of the clinical trial setting. Aside from challenges supplying the drug there is also a greater prescribing responsibility placed on the clinician to satisfy regulatory requirements^35^, in addition to managing unintended drug-drug interactions with coexisting treatment. Effective deployment in clinical practice should be accompanied by robust data to direct both penicillin and probenecid dosing and include both adult and paediatric populations.

### Strengths and limitations

This study brings gold standard analytical technologies to the fore for penicillin-V and probenecid PK, including protein binding characteristics. Using population pharmacokinetic modelling we have added the expected impact of probenecid on PTA for a range of penicillin-V doses. The strengths of this study are that free concentrations of penicillin-V and probenecid were directly measured reflecting the active fraction of the drug. This approach is preferred to estimating the free fraction based on prior knowledge or estimation which do not account for variation in binding. A crossover design was used to allow a direct comparison between individual visits thereby reducing the influence of interindividual PK variability. In this study we used *S. pneumoniae* as an example organism however the data can be rapidly applied to other microorganisms with expected susceptibility to penicillin-V, providing further dose structures for investigation.

This study also had limitations. Healthy volunteer studies may not mimic the target population given that patient PK are likely to be more variable. The data can be used to postulate dose regimens for further testing in a patient population with a view to understanding more about PK variability. We used a sparse sampling strategy with limited data points but combined this with rich data from a previous healthy volunteer study^20^. For logistical reasons we were unable to observe all dosing time-points that were taken by the participant at home. To minimise the risk of inaccurate dose scheduling that might affect pharmacokinetic modelling our participants completed a dosing card. The final dose taken prior to sampling was also observed in all cases.

## Conclusion

Addition of probenecid to penicillin was safe and well tolerated. Our PK data demonstrate a significant reduction in penicillin clearance when given alongside probenecid. Total and free probenecid concentrations remained stable in the first three hours of the dosing interval. This data will support the development of further studies to allow improved dosing structures for complex outpatient therapy or oral therapies where frequent administration is challenging. Probenecid also has a role in increasing target attainment for microorganisms with high MICs, to address potential penicillin supply shortages and maximising the effectiveness of other WHO “Access” agents.

### Supplementary Information

Supplementary Information.

## Data Availability

The datasets used and/or analysed during the current study are available from the corresponding author on reasonable request.
