# Supplementary material for: Towards pharmacokinetic boosting of phenoxymethylpenicillin (penicillin-V) using probenecid for the treatment of bacterial infections
Source: Sci Rep. 2024 Jul 21;14:16762. doi: 10.1038/s41598-024-67354-6 (PMC11271292; doi:10.1038/s41598-024-67354-6)
Supplement: Supplementary file 1 — Supplementary Information. [file 41598_2024_67354_MOESM1_ESM.pdf]

Towards pharmacokinetic boosting of phenoxymethylpenicillin (penicillin-V) using probenecid for the treatment of bacterial infections

Richard C Wilson, Alaa Riezk, Paul Arkell, Damien Ming, Ryan Armiger, Victoria Latham, Mark J Gilchrist, Anne-Grete Märtson, William W Hope, Alison H Holmes, Timothy M Rawson

Supplementary 1A. Pharmacokinetic structural model.

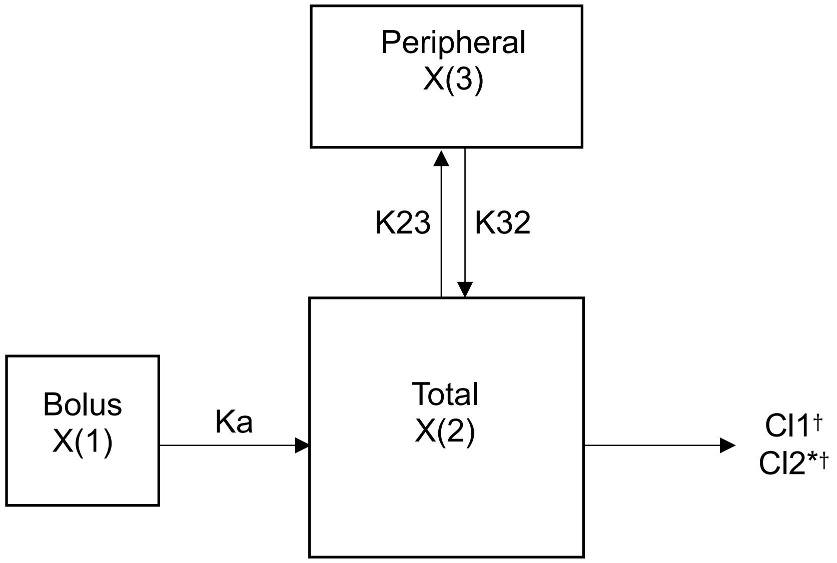

†Ke = (Cl1 * (1-PBC) + Cl2 * PBC) / V

*When administered alongside probenecid

Output

Y(1) = X(2)/V

Supplementary 1B. Parameter estimates for the pharmacokinetic model.

Parameter	Median	95% credibility Interval	Shrinkage (%)
Ka (hr^{-1})	1.154	0.942-1.356	5.28
V/F (L)	26.510	14.858-39.471	1.87
Cl1/F (L/hr)	63.148	54.757-68.229	1.75
Cl2/F (L/hr)	21.792	16.186-30.515	3.31
K23 (hr^{-1})	0.004	0.001-0.251	8.08
K32 (hr^{-1})	8.315	3.065-9.79	7.25

Supplementary 1C. Diagnostic plot for the final population pharmacokinetic model. a) Observed penicillin-V concentrations versus population-predicted penicillin-V concentration, b) Observed penicillin-V concentrations versus individual-predicted penicillin-V concentration. CI, confidence interval.

a)

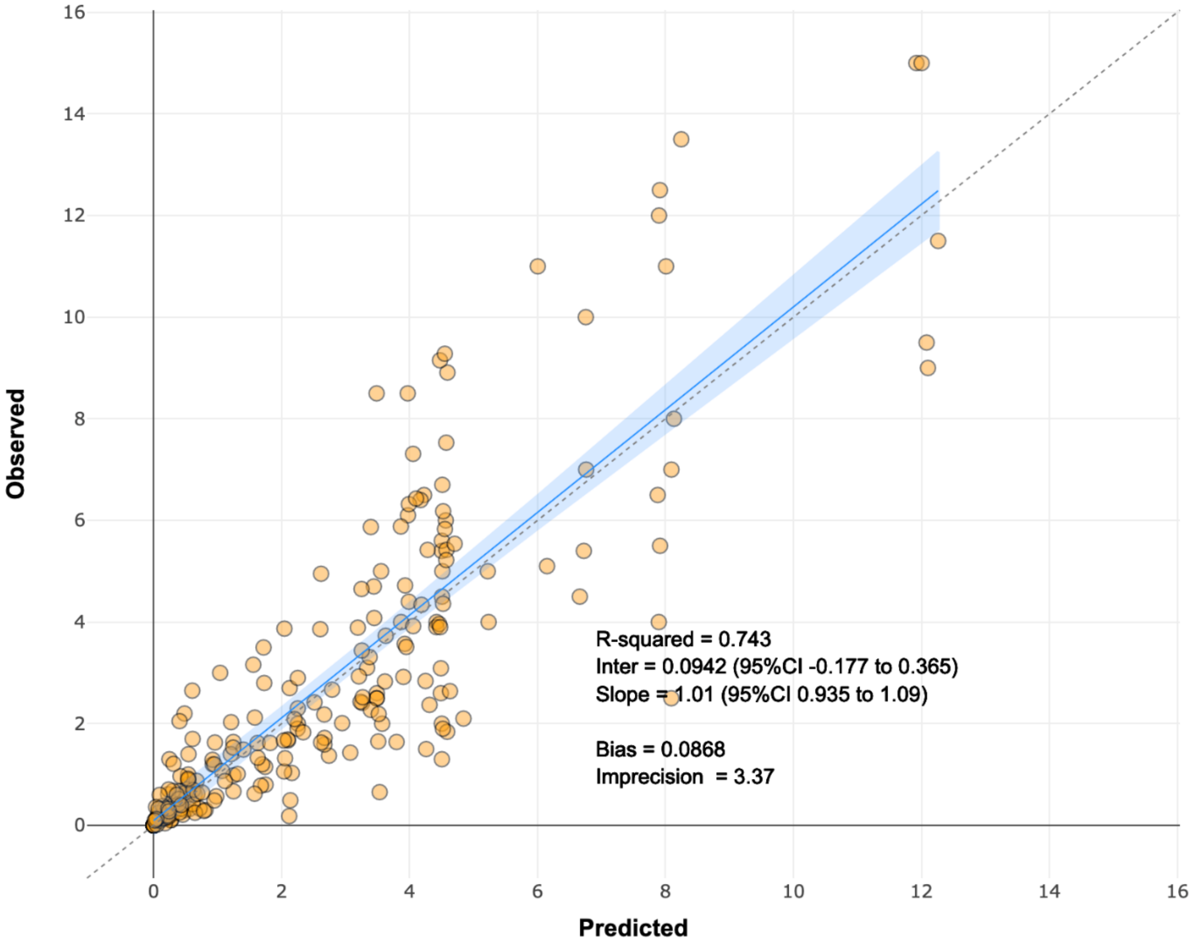

b)

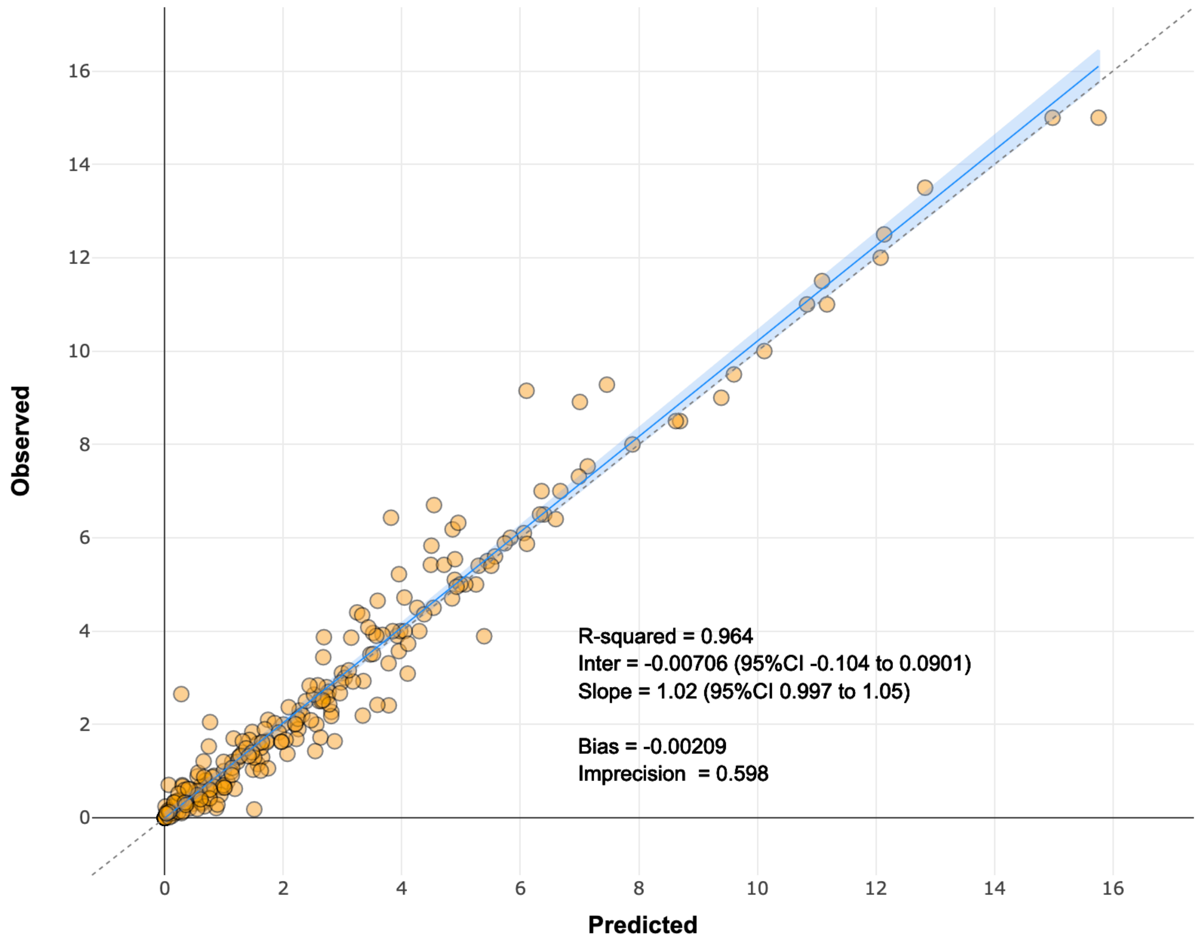

Supplementary 1D. Visual predictive check.

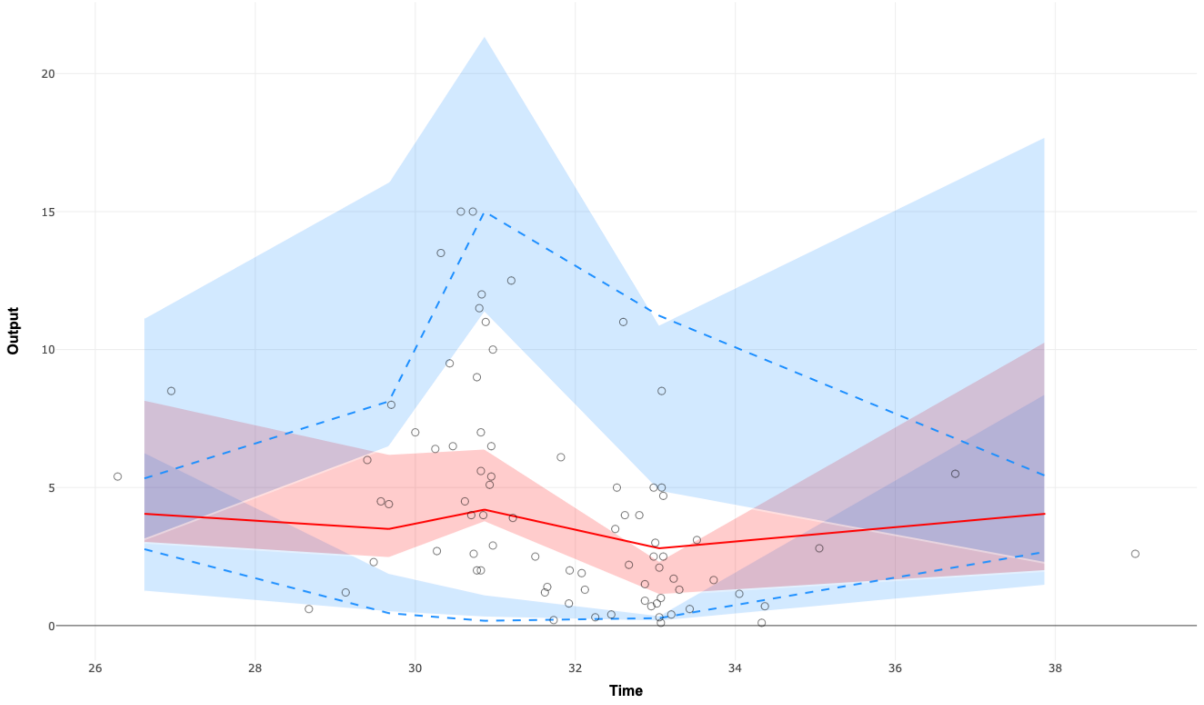

Supplementary: Protocol

ADDPROPEN STUDY

Addition of probenecid to penicillin-V therapy: an open label, cross-over study in healthy volunteers

<Version 2.2 23/07/2021>

MAIN SPONSOR: Imperial College London

FUNDERS: British Infection Association (BIA)

STUDY COORDINATION CENTRE: Imperial College London

NRES reference: **21/LO/0558**

IRAS Project ID: **299581**

EudraCT reference: **2021-002800-11**

Protocol authorised by:

Name & Role	Date	Signature
Timothy M Rawson	23/07/2021	

Study Management Group

Chief Investigator: Timothy Miles Rawson

Co-investigators: Richard Wilson
Alaa Riezk
Paul Arkell
Mark Gilchrist
Alison Holmes

Study Coordination Centre

For general queries, supply of study documentation, and collection of data, please contact:

Study Coordinator: Timothy Miles Rawson
Address: Imperial College London
Tel: 07891750151
E-mail: tmr07@ic.ac.uk
Web address: http://www.imperial.ac.uk/people/timothy.rawson07

Clinical Queries

Clinical queries should be directed to Timothy M Rawson who will direct the query to the appropriate person.

Sponsor

Imperial College London is the main research Sponsor for this study. For further information regarding the sponsorship conditions, please contact the Head of Regulatory Compliance at:

Research Governance and Integrity Team
Imperial College London and Imperial College Healthcare NHS Trust
Room 215, Level 2, Medical School Building
Norfolk Place
London, W2 1PG
Tel: 0207 594 9459

Funder

The British Infection Association (BIA) is funding this research.

This protocol describes the named study and provides information about procedures for entering participants. The protocol should not be used as a guide for the treatment of other participants; every care was taken in its drafting, but corrections or amendments may be necessary. These will be circulated to investigators in the study, but centres entering participants for the first time are advised to contact the trials centre to confirm they have the most recent version.

Problems relating to this trial should be referred, in the first instance, to the study coordination centre.
This trial will adhere to the principles outlined in the Medicines for Human Use (Clinical Trials) Regulations 2004 (SI 2004/1031), amended regulations (SI 2006/1928) and the International Conference on Harmonisation Good Clinical Practice (ICH GCP) guidelines. It will be conducted in compliance with the protocol, the Data Protection Act and other regulatory requirements as appropriate.

Table of Contents

1.	INTRODUCTION	13
1.1	BACKGROUND	13
2.	STUDY OBJECTIVES	13
3.	STUDY DESIGN	14
3.1	STUDY OUTCOME MEASURES	16
4.	PARTICIPANT ENTRY	16
4.1	PRE-RANDOMISATION EVALUATIONS	16
4.2	INCLUSION CRITERIA	16
4.3	EXCLUSION CRITERIA	16
4.4	WITHDRAWAL CRITERIA	17
5.	RANDOMISATION AND ENROLMENT PROCEDURE	17
5.1	RANDOMISATION OR REGISTRATION PRACTICALITIES	17
5.2	CONTRACEPTION REQUIREMENTS FOR FEMALE PARTICIPANTS	17
6.	TREATMENTS	18
6.1	TREATMENT ARMS	18
6.2	DOSE MODIFICATIONS FOR TOXICITY	19
6.3	PREMEDICATION	19
6.4	INTERACTION WITH OTHER DRUGS	19
6.5	DISPENSING AND ACCOUNTABILITY	19
6.6	SAFETY MONITORING ASSESSMENTS	19
7.	PHARMACOVIGILANCE	20
7.1	DEFINITIONS	20
7.2	CAUSALITY	21
7.3	REPORTING PROCEDURES	21
8.	ASSESSMENT AND FOLLOW-UP	23
8.1	INCIDENTAL FINDINGS	23
8.2	LOSS TO FOLLOW-UP	23
8.3	TRIAL CLOSURE	23
9.	STATISTICS AND DATA ANALYSIS	23
10.	MONITORING	24
10.1	RISK ASSESSMENT	24
10.2	MONITORING AT STUDY CENTRE	24
11.	REGULATORY ISSUES	24
11.1	CLINICAL TRIALS APPROVAL	24
11.2	ETHICS APPROVAL	24

11.3 CONSENT 24
11.4 CONFIDENTIALITY 25
11.5 INDEMNITY 25
11.6 SPONSOR 25
11.7 FUNDING 25
11.8 AUDITS AND INSPECTIONS 25
12. TRIAL MANAGEMENT 25
13. PUBLICATION POLICY 25
11. REFERENCES 25
APPENDIX 1. SUMMARY OF STUDY 27
APPENDIX 2. SUMMARY OF INVESTIGATIONS, TREATMENTS AND ASSESSMENTS 28

GLOSSARY OF ABBREVIATIONS

FBC	Full Blood Count
Hb	Haemoglobin
HBV	Hepatitis B Virus
HCV	Hepatitis C Virus
HIV	Human Immunodeficiency Virus
LFT	Liver Function Tests
PD	Pharmacodynamics
PK	Pharmacokinetics
QDS	Four times a day / 6-hourly
SmPC	Summary of medicinal product characteristics
ULN	Upper Limit of Normal
%fT>MIC	Percentage time the free drug concentration spends above the minimum inhibitory concentration

KEYWORDS
Beta-lactams, probenecid, pharmacokinetic-pharmacodynamic targets, healthy volunteer study, antimicrobial optimisation.

STUDY SUMMARY

**TITLE**	Addition of probenecid to penicillin-V therapy: a randomised, open label, cross-over study in healthy volunteers
**DESIGN**	Randomised, open-label, cross-over study.
**HYPOTHESIS**	Addition of probenecid to oral phenoxymethylpenicillin (penicillin-V) has a clinically relevant influence of pharmacokinetic-pharmacodynamic (PK-PD) target attainment.
**AIMS**	To describe the influence of concomitant probenecid administration on penicillin-V PK in healthy-volunteers.<br><br>Estimate the influence probenecid driven PK variation on penicillin-V PK-PD targets (i.e. free time over the minimum inhibitory concentration, fT>MIC).<br><br>Develop a population PK-PD model that can support the design of future prospective studies exploring optimal methods for penicillin-V dose optimisation.
**OUTCOME MEASURES**	Free, unbound serum drug concentration at 45 & 180 minutes post antibiotic dose.
**POPULATION**	50 healthy adult volunteers recruited to attend Imperial College Clinical Research Facility, Hammersmith Hospital, UK.
**ELIGIBILITY**	Healthy volunteers over 18 years old.
**TREATMENT**	Addition of probenecid to penicillin-V.
**DURATION**	1 year (12 months)

REFERENCE DIAGRAM

See appendix 1.

1. INTRODUCTION

1.1 BACKGROUND

Probenecid, p-(di-*n*-propylsulfamyl)-benzoic acid, was developed in 1949 with the purpose of decreasing the renal clearance of penicillin.[1] The development of probenecid stemmed from the requirement to ration supplies of penicillin during World War II. Its mechanism of action is thought to be to the competitive inhibition of organic anion transporters, which are responsible for the excretion of organic agents, such as penicillin.[2] Reduction in renal clearance of penicillin with probenecid demonstrated significant increases in serum concentrations, meaning that lower doses of drug were required for similar pharmacokinetic-pharmacodynamic (PK-PD) target attainment. Probenecid's influence on penicillin clearance became mainly academic in the post-war era as our capability to produce more diverse, cheaper, and safer beta-lactam antibiotics rapidly expanded.[1] Probenecid remains a recommended adjunct in the management of some sexually transmitted diseases to support therapeutic target attainment in compartments, such as cerebrospinal fluid.[3] However, its potential important broader role in preserving the effectiveness of beta-lactams through the optimisation of beta-lactam PK needs to be considered.

The World Health Organisation (WHO) AWaRe criteria require narrow spectrum antimicrobials, such as the penicillins, to be available in appropriate type, dose, and duration to treat common infections.[4] With increasing drug-resistance within common causative organisms, such as in streptococcal infections, new methods to optimise the delivery of *Access* agents and protect the use of broader *Watch* and *Reserve* antimicrobials is required.[4] It is not always possible to simply prescribe higher doses of an antibiotic to overcome problems such as increasing drug-resistance. In some instances, oral drug absorption or gastrointestinal side effects associated with high doses limit escalation of therapy. Some agents are not licenced for use at doses required to obtain acceptable PK-PD targets for high minimum-inhibitory concentration (MIC) organisms. Some settings, such as outpatient parenteral antibiotic therapy (OPAT) do not allow for multiple daily dosing schedules of an intravenous drug, meaning that an oral or once daily alternative intravenous option is required. Probenecid offers a means of optimising the delivery of oral and intravenous antimicrobial therapy through manipulation of beta-lactam PK without significant changes to antimicrobial dosing.

1.2 RATIONALE FOR CURRENT STUDY

This study aims to build on previous work characterising the PK of penicillin-V to explore the potential impact of probenecid on PK-PD target attainment. Achievement of the aims of this study would provide data to support the design of experimental studies exploring the clinical impact of probenecid on treatment outcomes and also provide a rationale for exploration of probenecid's effects on a larger number of beta-lactam antibiotics.

Hypothesis: Addition of probenecid to oral phenoxymethylpenicillin (penicillin-V) has a clinically relevant effect on pharmacokinetic-pharmacodynamic (PK-PD) target attainment.

2. STUDY OBJECTIVES

1. To describe the influence of concomitant probenecid administration on penicillin-V PK in healthy-volunteers.

2. Estimate the influence probenecid driven PK variation on penicillin-V PK-PD targets (i.e. free time over the minimum inhibitory concentration, fT>MIC).

3. Develop a population PK-PD model that can support the design of future prospective studies exploring optimal methods for penicillin-V dose optimisation.

3. STUDY DESIGN

This study will link rich penicillin-V pharmacokinetic data from a prior healthy volunteer study[5] to sparse PK data collected prospectively as part of a randomised, open-label, cross-over study outlined below. This will support the development of a population PK model that describes probenecid's influence on renal clearance of penicillin-V as a covariate.

Population: 50 healthy adult volunteers recruited to attend Imperial College Clinical Research Facility, Hammersmith Hospital, UK.
Design: Randomised, open-label, cross-over study.
Intervention: Oral penicillin-V plus oral probenecid.
Comparator: Oral penicillin-V.
Outcome: Free, unbound drug concentration at 45 & 180 minutes post antibiotic dose.
Eligibility: Healthy volunteers over 18 years old.
Inclusion: Adult, eGFR > 90, previously received penicillin-based antibiotic.
Exclusion: Penicillin or probenecid allergy, eGFR < 90, pregnant (or likely to become), anaemic, abnormal liver function, lack capacity, history of gout or uric acid kidney stones, G6PD deficiency, symptoms indicative of active infection, medications that interact with probenecid. Recent involvement in other research (within prior 3 months).
Randomisation:
Simple randomisation will be performed aiming for 50% of individuals to commence on intervention and 50% comparator arm of the study.
Blinding: This is an unblinded study.

Sample size: 50 participants.
Using sparse PK sampling in a cross-over design; previous estimates have suggested that a sample size of at least 13 participants are required to detect a binary covariate (i.e. 30% proportional increase in elimination clearance) with at least 80% statistical power and a significance of p=0.05. Prior studies, using different methodological approaches and penicillin-based antimicrobials have suggested that probenecid has approximately 30-40% effect on drug clearance.

Statistical analysis:
PK modelling of data will be undertaken using Pmetrics in R. Non-linear mixed effects modelling will be used to estimate the posterior estimates for individual PK. Probenecid exposures influence on clearance will be explored as a covariate in the model.

Probability of target attainment (40% fT>MIC) for common organisms (e.g. streptococci) will be estimated. Descriptive statistics will be applied to the estimated population parameters and simulated PTAs to facilitate comparison of the data.

Summary of method:
Participants will be screened and consented to attend Imperial College Clinical Research Facility (CRF) at Hammersmith Hospital on two study visits, at least 7 days apart. For one visit (randomised), participants will be required to take penicillin-V only. For their other visit, they will take penicillin-V plus probenecid at standard recommended dose. Prior to the study visits, participants may be required to have taken 36-hours of penicillin +/- probenecid, documenting this in a dosing diary. On arrival at the CRF, the participant will take an observed dose of penicillin +/- probenecid. They will undergo blood draw via needle phlebotomy or a cannula (participant choice) at 45 and 180 minutes post the observed. Samples will be spun down and frozen at -80°C. They will subsequently be analysed using an in-house HPLC-MS/MS methodology to determine total and free-unbound drug concentration.

For analysis, data from this study will be pooled with rich PK data from a prior study that assessed plasma concertation of penicillin-V in healthy volunteers. Pmetrics in R will be used to model the data looking to explore the effect of probenecid on clearance of free-penicillin-V. Probability of target attainment for streptococci species will also be estimated to evaluate the potential clinical impact of the addition of probenecid to routine penicillin-V use. Rich PK data for intravenous benzylpenicillin will be used to estimate PK-PD target attainment and PTAs for intravenous formulations, allowing direct comparison of oral and IV regimes.

Figure. Total plasm penicillin concentration – time profiles for healthy volunteers receiving oral penicillin-V.

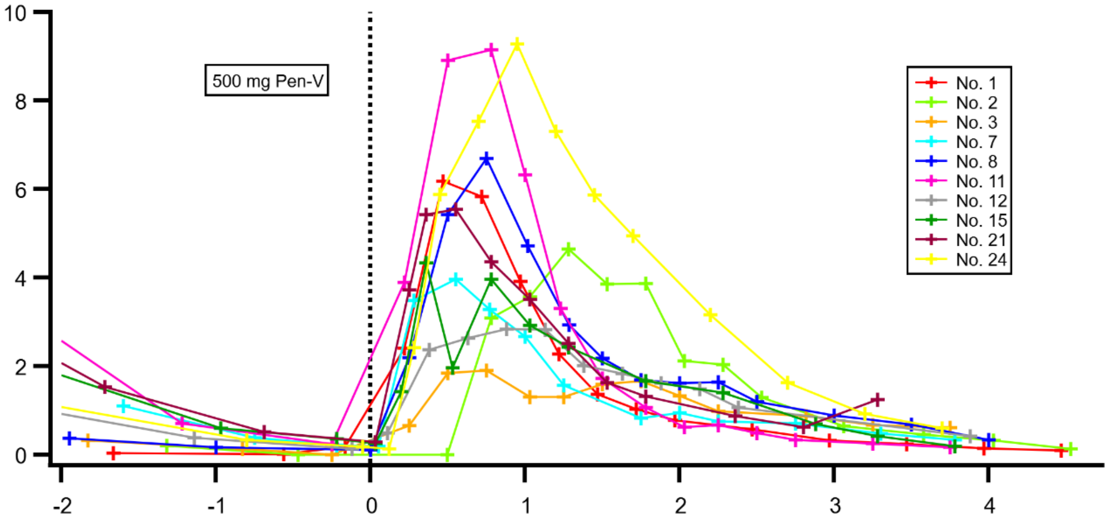

Medication used in the study:
Penicillin-V and probenecid used in this study will be prescribed by a qualified doctor. It will be supplied by Imperial College Healthcare NHS Trust pharmacy.

Probenecid will be dosed at 500mg four-time a day (QDS) for all participants.
Note: probenecid is not licenced for use in the United Kingdom. It is licenced in the EU. The dose selected is based on the probenecid summary of product characteristics, known doses used safely in the treatment of neuro-syphilis with benzylpenicillin as part of British STI treatment guidance, and is in line with published literature and guidelines for unlicensed use in the treatment of infection within UK and international organisations. Probenecid will be sourced from BioPhausia AB, Sweeden via Imperial College Healthcare NHS Trust Pharmacy. Dosing will be in line with their SmPC.

Penicillin-V within the study will be dosed as follows for participants:

Group 1 (n=24)	500mg QDS penicillin-V
Group 2 (n=13)	250mg QDS penicillin-V
Group 3 (n=13)	750mg QDS penicillin-V

Dose escalation in increments of 250mg has been selected to provide a wide safety margin even at higher doses. Probenecid is estimated to reduce penicillin clearance by approximately 30%. Given that the maximum licenced dose of penicillin-V in the UK is 4g / day, we estimate that for a 30% reduction in clearance, 750mg QDS should remain within this margin of safety. Furthermore, given that doses of intravenous penicillin achieve much greater concentrations with limited side effects, we do not anticipate issues with toxicity within this study.

Both drugs will be dispensed by Imperial College Hospital NHS Trust pharmacy with labelling performed in-house in alignment with Annex 13 under the Reg 37 hospital exemption.

3.1 STUDY OUTCOME MEASURES

Primary outcome measure:

1. Total and unbound penicillin-V concentrations at 45 and 180 minutes post observed penicillin-V dose with and without probenecid.

Secondary outcome:

1. Probability of target attainment for common infections treated with penicillin (e.g. *Streptococcus spp.*) will be estimated.
2. PK-PD model describing the influence of probenecid on penicillin-V in healthy volunteers.

Other documented outcomes:

1. Reported adverse events associated with penicillin-V +/- probenecid.

4. PARTICIPANT ENTRY

4.1 PRE-RANDOMISATION EVALUATIONS

Pre-registration evaluation will include:
- Clinical assessment
- Confirmation of prior penicillin use and no documented penicillin allergy.
- Blood tests including FBC, LFT, Renal Profile, Blood Borne Viral Screen (HIV/HBV/HCV).
- Pregnancy test in female participants.

4.2 INCLUSION CRITERIA

- Adult healthy volunteers (>18 years old).
- Previously taken penicillin-based antibiotics without allergic response.
- Estimated Glomerular Filtration Rate (eGFR) > 90.

4.3 EXCLUSION CRITERIA

- Lacking capacity to consent.
- Documented allergy to penicillin, other beta-lactam antibiotics, or probenecid.
- History of G6PD Deficiency.
- Known blood dyscrasias.
- Anaemia (Hb < 12g/dL female, 13g/dL males).
- Abnormal liver function (ALT, AST, ALP or bilirubin > ULN).
- eGFR < 90.
- Pregnant or likely to become pregnant during study period.
- Breastfeeding women.
- Symptoms consistent with active infection.
- History of gout or uric acid kidney stones.
- Taking regular medication that may interact with probenecid including, but not limited to methotrexate, lorazepam, acetaminophen, oral hypoglycaemic medication, sulfa containing drugs, non-steroidal anti-inflammatory drugs.
- History of evidence of any medical, neurological, or psychological condition that would expose the subject to an undue risk of a significant adverse event or interfere with study assessments during the course of the trial as determined by the clinical judgement of the investigator.
- Recent involvement in other research (within prior 3 months).

4.4 WITHDRAWAL CRITERIA

Participants can withdraw at any point from the study without providing a reason for their decision. If they choose to withdraw after entry into the study any samples provided to this point (with their consent) will be retained for use in the study.

If a participant develops any exclusion criteria after enrolment, they will be withdrawn from the study immediately.

Study stopping rules:

If information were to arise during the course of this study that would negate the results or suggest any safety concerns, the study will be stopped immediately with participants withdrawn and an update provided to them explaining the rationale for this.

IMP treatment will be discontinued individually if a study participant presents a severe or serious adverse event considered related to the study IMPs.

The trial will be halted if any of the following is observed:

- A serious adverse reaction (i.e. a serious adverse event considered at least possibly related to the IMP administration) in one subject.
- Severe adverse reactions (i.e. severe adverse events considered as, at least, possibly related to the IMP administration) in two subjects, independent of within or not within the same system-organ-class.

If the trial is halted due to any of the study stopping rules detailed above, the trial will only be re-started after regulatory authority approval via a substantial amendment.

5. RANDOMISATION AND ENROLMENT PROCEDURE

5.1 RANDOMISATION OR REGISTRATION PRACTICALITIES

Pre-randomisation screening will require clinical assessment, checking of eligibility, pregnancy testing if female, and screening blood tests (FBC, LFT, Renal Profile, HIV, HBV, & HCV).

Randomisation will be performed using simple randomisation. This will aim for 50% of individuals to commence on intervention and 50% comparator arm of the study. A randomisation list will be generated prior to commencement of the study. Participants will then be added sequentially onto this list as they enter the study.

This study will be unblinded.

5.2 CONTRACEPTION REQUIREMENTS FOR FEMALE PARTICIPANTS

Probenecid crosses the placental barrier and appears in cord blood, and the use of this drug in patients that are women of childbearing potential requires that the anticipated benefit be weighed against possible hazards. As this clinical trial is in healthy volunteers whom are not deriving any benefit by taking part in the trial, female participants must follow adequate contraception advice during the full participation in the trial.

This trial follows the contraception guidance for female participants set out in the Clinical Trial Facilitation Group (CTFG) guidance on ‘Recommendations related to contraception and pregnancy testing in clinical trials’.

A woman of childbearing potential (WOCBP) is defined as a "*female participant that is fertile, following menarche and until becoming post-menopausal unless permanently sterile. Permanent sterilisation methods include hysterectomy, bilateral salpingectomy and bilateral oophorectomy. A postmenopausal state is defined as no menses for 12 months without an alternative medical cause. A high follicle stimulating hormone (FSH) level in the postmenopausal range may be used to confirm a postmenopausal state in women not using hormonal contraception or hormonal replacement therapy (HRT). However, in the absence of 12 months of amenorrhea, confirmation with more than one FSH measurement is required*".

For WOCBP, acceptable methods of contraception must be used for the total duration of enrolment in the trial. Acceptable methods of contraception to be included within this study are defined within section 4 of the CTFG guidance as:

Acceptable and highly effective methods of birth control

- Combined hormonal contraception (oestrogen and progesterone containing) associated with inhibition of ovulation (oral, intravaginal, or transdermal).
- Progesterone only hormonal contraception associated with the inhibition of ovulation (oral, injectable, or implantable).
- Intrauterine device (IUD).
- Intrauterine hormone-releasing system (IUS).
- Bilateral tubal occlusion.
- Vasectomised partner.
- Sexual abstinence*

*NB: sexual abstinence is considered a highly effective method only if defined as refraining from heterosexual intercourse during the entire period of risk associated with the study treatments. The reliability of sexual abstinence needs to be evaluated in relation to the duration of the clinical trial (estimated at 48 hours for each dosing interval) and the preferred and usual lifestyle of the subject. Clear documentation of this decision must be made at the time of screening.

Acceptable but not considered as highly effective methods of birth control

- Progesterone-only oral hormonal contraception, where inhibition of ovulation is not the primary mode of action.
- Male or female condom with or without spermicide.
- Cap, diaphragm, or sponge with spermicide.
- Double barrier methods (e.g. male condom with cap, diaphragm, or sponge with spermicide) are also considered as acceptable, but not highly effective methods of birth control.

6. TREATMENTS

6.1 TREATMENT ARMS

All treatments (penicillin-V and probenecid) used within this study will be supplied by Imperial College Healthcare NHS Trust pharmacy.

Probenecid will be dosed at 500mg QDS for all participants. It will be supplied by Imperial College Healthcare NHS Trust pharmacy. The formulation will be tablet form (500mg tablets).

Penicillin-V will be dosed as follows for participants:

Group 1 (n=24)	500mg QDS penicillin-V
Group 2 (n=13)	250mg QDS penicillin-V

Group 3 (n=13)	750mg QDS penicillin-V

It will be supplied by Imperial College Healthcare NHS Trust pharmacy. The formulation will be tablet form (250mg tablets).

All participants will be required to commence treatment 36 hours prior to the CRF visit. They will take a maximum of 6 doses of penicillin-V +/- probenecid prior to attending the CRF. They will then take a further observed dose of penicillin-V +/- probenecid during the CRF visit (at time-point 0 minutes). The requirement for 6 doses prior to attending the CRF visit is to ensure that drug concentration (penicillin-V +/- probenecid) is at steady state (normally requires approximately 5 doses) prior to drug level sampling. In total, throughout the study (both visits), participants will take a maximum of 14 doses of penicillin-V and 7 doses of probenecid. This treatment interval has been selected as it is the shortest feasible duration to ensure patients arrive for the study visit at steady-state, whilst minimising the potential risks to safety in terms of drug interactions and adverse events.

6.2 DOSE MODIFICATIONS FOR TOXICITY

No dose reductions are anticipated in this study. Participants will be required to have normal renal function. The dose of penicillin-V is within its licensed range. The dose of probenecid used is widely used in clinical practice with minimal reported side effects.

Dose escalation in increments of 250mg has been selected to provide a wide safety margin even at higher doses. Probenecid is estimated to reduce penicillin clearance by approximately 30%. Given that the maximum licenced dose of penicillin-V in the UK is 4g / day, we estimate that for a 30% reduction in clearance, 750mg QDS should remain within this margin of safety. Furthermore, given that doses of intravenous penicillin achieve much greater concentrations, we do not anticipate issues with toxicity within this study.

6.3 PREMEDICATION

No premedication will be required as part of this study.

6.4 INTERACTION WITH OTHER DRUGS

Participants will be informed of potential interactions of probenecid and other drugs. These include but a not limited to: methotrexate, lorazepam, acetaminophen, oral hypoglycaemic medication, sulfa containing drugs, non-steroidal anti-inflammatory drugs.

Participants will be asked not to take other medications if possible during the dosing visits. If they require paracetamol, they will be advised to take 500mg QDS maximum.

6.5 DISPENSING AND ACCOUNTABILITY

All medications used within this study will be supplied via the NHS suppliers which will be overseen by Imperial College Healthcare NHS Trust Clinical Trials Pharmacy team. The investigation medicinal product (IMP) will be used in line with the summary of medicinal product characteristics obtained from the EU-licensed supplier. All medications will be prescribed by a physician within the research team at the doses required per participant.

IMP 1: Probenecid (tablet form) – EU market authorisation BioPhausia AB (MA number: 4522)
IMP 2: Penicillin-V (tablet form) – UK market authorisation Sandoz BmB (MA number: PL 04520/0005)

6.6 SAFETY MONITORING ASSESSMENTS

If there are concerns with potential participant safety as defined by investigator criteria or clinical indication at any time during the study a safety monitoring assessment will be performed by a clinician within the study team. This may include physical examination, blood testing, and measurement of participant vital signs.

7. Pharmacovigilance

7.1 DEFINITIONS

Adverse Event (AE): any untoward medical occurrence in a patient or clinical trial subject administered a medicinal product and which does not necessarily have a causal relationship with this treatment. *An AE can therefore be any unfavourable and unintended sign (including an abnormal laboratory finding), symptom, or disease temporally associated with the use of an investigational medicinal product (IMP), whether or not considered related to the IMP.*

Based on the investigator judgement, an AE will be defined as:

Mild: An event that does not affect the activities of daily living. It is easily tolerated. The effect is mild and does not impact the individual.

Moderate: Affects normal activities of daily living. Affects the ability of one to carry out tasks which they normally do. It causes discomfort and distress to the individual.

Severe: An event that prevents normal daily activities.

Adverse Reaction (AR): all untoward and unintended responses to an IMP related to any dose administered. *All AEs judged by either the reporting investigator or the sponsor as having reasonable causal relationship to a medicinal product qualify as adverse reactions. The expression reasonable causal relationship means to convey in general that there is evidence or argument to suggest a causal relationship.*

Unexpected Adverse Reaction: an AR, the nature or severity of which is not listed in the reference safety information (RSI) e.g. list of expected medical events within investigator's brochure for an unapproved investigational product or section 4.8 of the summary of product characteristics (SmPC) for an authorised product. *When the outcome occurs this adverse reaction should be considered as unexpected. Side effects documented in the SmPC which occur in a more severe form than anticipated are also considered to be unexpected.*

Serious Adverse Event (SAE) or **Serious Adverse Reaction:** any untoward medical occurrence or effect that at any dose:

- **Results in death.**
- **Is life-threatening** – *refers to an event in which the subject was at risk of death at the time of the event; it does not refer to an event which hypothetically might have caused death if it were more severe.*
- **Requires hospitalisation, or prolongation of existing inpatients' hospitalisation.**
- **Results in persistent or significant disability or incapacity.**
- **Is a congenital anomaly or birth defect.**

Medical judgement should be exercised in deciding whether an AE/AR is serious in other situations. Important AE/ARs that are not immediately life-threatening or do not result in death or hospitalisation but may jeopardise the subject or may require intervention to prevent one of the other outcomes listed in the definition above, should also be considered serious.

Suspected Unexpected Serious Adverse Reaction (SUSAR): any suspected adverse reaction related to an IMP that is both unexpected and serious.

7.2 CAUSALITY

Most adverse events and adverse drug reactions that occur in this study, whether they are serious or not, will be expected treatment-related toxicities due to the drugs used in this study. The assignment of the causality should be made by the investigator responsible for the care of the participant using the definitions in the table below.

If any doubt about the causality exists the local investigator should inform the study coordination centre who will notify the Chief Investigators. The pharmaceutical companies and/or other clinicians may be asked to advise in some cases.

In the case of discrepant views on causality between the investigator and others, all parties will discuss the case. In the event that no agreement is made, the MHRA will be informed of both points of view.

Relationship	Description
**Unrelated**	There is no evidence of any causal relationship
**Unlikely**	There is little evidence to suggest there is a causal relationship (e.g. the event did not occur within a reasonable time after administration of the trial medication). There is another reasonable explanation for the event (e.g. the participant's clinical condition, other concomitant treatment).
**Possible**	There is some evidence to suggest a causal relationship (e.g. because the event occurs within a reasonable time after administration of the trial medication). However, the influence of other factors may have contributed to the event (e.g. the participant's clinical condition, other concomitant treatments).
**Probable**	There is evidence to suggest a causal relationship and the influence of other factors is unlikely.
**Definitely**	There is clear evidence to suggest a causal relationship and other possible contributing factors can be ruled out.
**Not assessable**	There is insufficient or incomplete evidence to make a clinical judgement of the causal relationship.

7.3 REPORTING PROCEDURES

All adverse events should be reported. Depending on the nature of the event the reporting procedures below should be followed. Any questions concerning adverse event reporting should be directed to the study coordination centre in the first instance. A flowchart is given below to aid in the reporting procedures. All AE and SAEs must be recorded from the time of consent, until the end of the last study visit.

7.3.1 Non serious AR/AEs

All such toxicities, whether expected or not, should be recorded in the toxicity section of the relevant case report form and sent to the study coordination centre within one month of the form being due.

7.3.2 Serious AR/AEs

Fatal or life threatening SAEs and SUSARs should be reported on the day that the local site is aware of the event. The SAE form asks for nature of event, date of onset, severity, corrective therapies given, outcome and causality (i.e. unrelated, unlikely, possible, probably, definitely). The responsible investigator should sign the causality of the event. Additional information should be sent within 5 days if the reaction has not resolved at the time of reporting.

SAEs
An SAE form should be completed and faxed to the study coordination centre for all SAEs within 24 hours.

SUSARs
In the case of suspected unexpected serious adverse reactions, the staff at the site should:

Complete the SAE case report form & send it immediately (within 24 hours,), signed and dated to the study coordination centre together with relevant treatment forms and anonymised copies of all relevant investigations.

Or

Contact the study coordination centre by phone and then send the completed SAE form to the study coordination centre within the following 24 hours as above.

The study coordination centre will notify the MHRA, REC and the Sponsor of all SUSARs occurring during the study according to the following timelines; fatal and life-threatening within 7 days of notification and non-life threatening within 15 days. All investigators will be informed of all SUSARs occurring throughout the study.

Local investigators should report any SUSARs and /or SAEs as required by their Local Research Ethics Committee and/or Research & Development Office.

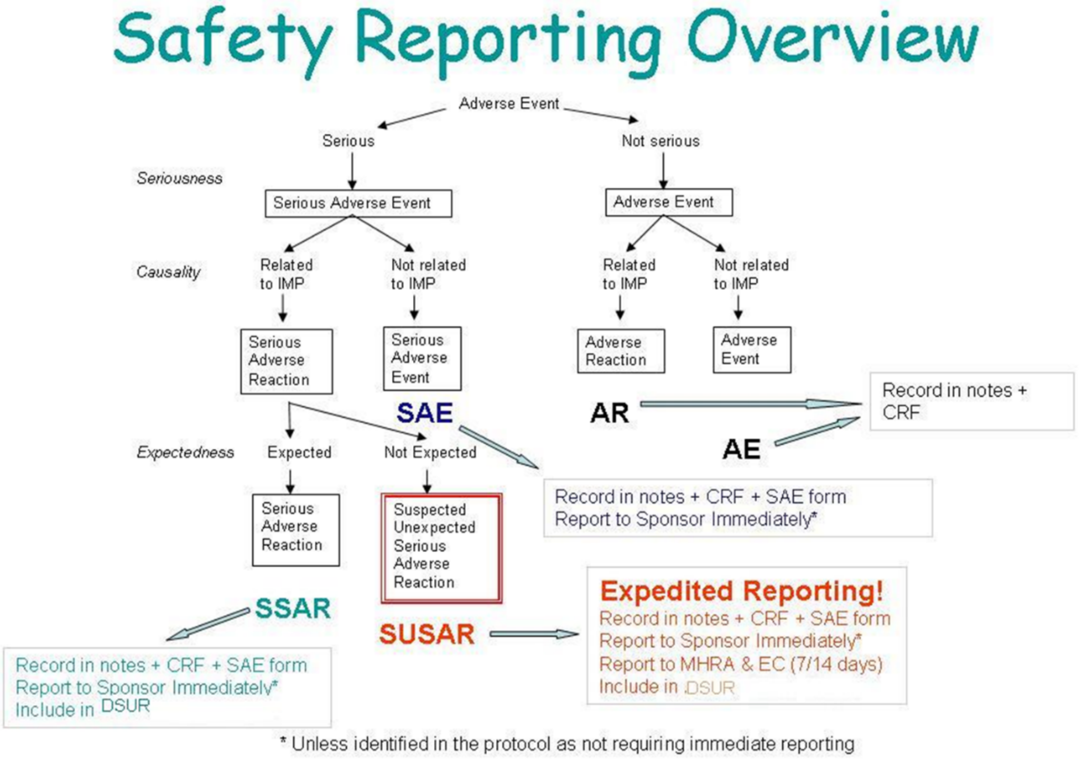

Contact details for reporting SAEs and SUSARs
RGIT.ctimp.team@imperial.ac.uk
CI email (and further details below).
Please send SAE forms to: tmr07@ic.ac.uk

Tel: 07891750151 (Mon to Fri 09.00 – 17.00)

8. ASSESSMENT AND FOLLOW-UP

After the second CRF visit there will be no further follow up of participants required. They will have completed their enrolment in the study at this time.

The end of the study will be defined as when all 50 participants entered into the study have completed two planned study visits.

8.1 INCIDENTAL FINDINGS

Incidental findings that arise as part of the study will be discussed with the patient. Permission will be sought to contact the patients GP to inform them of these findings.

8.2 LOSS TO FOLLOW-UP

No follow up is required post the second dosing visit to the Imperial CRF.

8.3 TRIAL CLOSURE

The definition for the end of the trial is when the last participant has completed their last visit. This is anticipated to be at participant number 50.

9. STATISTICS AND DATA ANALYSIS

Sample size: 50 participants.
Using sparse PK sampling in a cross-over design; previous estimates have suggested that a sample size of at least 13 participants are required to detect a binary covariate (i.e. 30% proportional increase in elimination clearance) with at least 80% statistical power and a significance of p=0.05. Prior studies, using different methodological approaches and penicillin-based antimicrobials have suggested that probenecid has approximately 30-40% effect on drug clearance.

Statistical analysis:
PK modelling of data will be undertaken using Pmetrics in R. Non-linear mixed effects modelling will be used to estimate the posterior estimates for individual PK. Probenecid exposures influence on clearance will be explored as a covariate in the model.

Probability of target attainment (40% fT>MIC) for common organisms (e.g. streptococci) will be estimated. Descriptive statistics will be applied to the estimated population parameters and simulated PTAs to facilitate comparison of the data.

Randomisation:
Simple randomisation will be performed using the randomizeR package in R. Randomisation will be performed prior to commencement of recruitment. This will be stored on a central database with participants allocated and randomised sequentially upon entry into study visit 1.

Interim analysis plan:

Formal interim analysis will be undertaken by members of the research team after every 10 participants have completed two study visits. Drug concentration data and reported side effects / adverse events will be evaluated.

Given the unblinded nature of this study, informal analysis will be iterative with researchers monitoring documented side effects / safety reporting per-participant visit to the CRF.

Data and all appropriate documentation will be stored for a minimum of 10 years after the completion of the study, including the follow-up period.

10. Monitoring

10.1 RISK ASSESSMENT

The study has been reviewed by Imperial Clinical Research Facility (CRF) Protocol Review Board (PRB). A risk assessment and management plan for CTIMP has been completed with the study scoring low risk on risk assessment.

10.2 MONITORING AT STUDY CENTRE

Routine monitoring will be performed for all participants throughout study visits. This will include data entry checking, consent form checking, and ensuring that all required data are input at the time of each participant visit. Transport and analysis logs for the processing, storage, and analysis of plasma samples will be kept to ensure that any abnormal results can be reviewed.

11. REGULATORY ISSUES

11.1 CLINICAL TRIALS APPROVAL

This study has Clinical Trials Authorisation from the UK Competent Authority; MHRA.
Reference: 2021-002800-11.

11.2 ETHICS APPROVAL

The Study Coordination Centre has obtained approval from the Riverside Regional Ethics Committee (ref: 21/LO/0558) and Health Regulator Authority (HRA). The study must also receive confirmation of capacity and capability from each participating NHS Trust before accepting participants into the study or any research activity is carried out. The study will be conducted in accordance with the recommendations for physicians involved in research on human subjects adopted by the 18th World Medical Assembly, Helsinki 1964 and later revisions.

11.3 CONSENT

Consent to enter the study must be sought from each participant only after a full explanation has been given, an information leaflet offered and time allowed for consideration. Signed participant consent should be obtained. The right of the participant to refuse to participate without giving reasons must be respected. After the participant has entered the trial the clinician remains free to give alternative treatment to that specified in the protocol at any stage if he/she feels it is in the participant's best interest, but the reasons for doing so should be recorded. In these cases the participants remain within the study for the purposes of follow-up and data analysis. All participants are free to withdraw at any time from the protocol treatment without giving reasons and without prejudicing further treatment.

11.4 CONFIDENTIALITY

Participants' identification data will be required for the registration process. The Study Coordination Centre will preserve the confidentiality of participants taking part in the study and is registered under the Data Protection Act.

11.5 INDEMNITY

Imperial College London holds negligent harm and non-negligent harm insurance policies which apply to this study.

11.6 SPONSOR

Imperial College London will act as the main Sponsor for this study. Delegated responsibilities will be assigned to the NHS trusts taking part in this study

11.7 FUNDING

The British Infection Association (BIA) are funding this study.

Reimbursement of participants travel costs will be offered at up to £50.00 per participant per study visit. Reimbursement cannot be provided as part of screening visits.

11.8 AUDITS AND INSPECTIONS

The study may be subject to inspection and audit by Imperial College London/Imperial College Healthcare NHS Trust (delete as applicable) under their remit as Sponsor, the Study Coordination Centre and other regulatory bodies to ensure adherence to GCP.

12. Trial Management

A Trial Management Group (TMG) will be appointed and will be responsible for overseeing the progress of the trial. The day-to-day management of the trial will be co-ordinated through the Centre for Antimicrobial Optimisation (Dr Timothy M Rawson).

13. PUBLICATION POLICY

The results of the study will be published within 12 months of study completion in a peer-reviewed journal. Preliminary results may also be presented at national and international academic research meetings, including ECCMID 2022 and FIS 2022.

All publications and presentations relating to the study will be authorised by the Trial Management Group. The first publication of the trial results will be in the name of the Trial Management Group, if this does not conflict with the journal's policy. If there are named authors, these will include at least the trial's Chief Investigator, Statistician and Trial Coordinator. Members of the TMG and the Data Monitoring Committee will be listed and contributors will be cited by name if published in a journal where this does not conflict with the journal's policy. Authorship of parallel studies initiated outside of the Trial Management Group will be according to the individuals involved in the project but must acknowledge the contribution of the Trial Management Group and the Study Coordination Centre.

11. REFERENCES

1. Robbins N, Koch SE, Tranter M, Rubinstein J. The History and Future of Probenecid. *Cardiovasc Toxicol* 2012; **12**: 1–9.

2. Maeda K, Tian Y, Fujita T, Ikeda Y, Kumagai Y, Kondo T *et al.* Inhibitory effects of p-aminohippurate and probenecid on the renal clearance of adefovir and benzylpenicillin as probe drugs for organic anion transporter (OAT) 1 and OAT3 in humans. *Eur J Pharm Sci* 2014; **59**: 94–103.

3. Frieden TR, Harold Jaffe DW, Rasmussen SA, Leahy MA, Martinroe JC, Spriggs SR *et al.* Morbidity and Mortality Weekly Report Sexually Transmitted Diseases Treatment Guidelines, 2015. https://www.cdc.gov/std/tg2015/default.htm

4. Sharland M, Pulcini C, Harbarth S, Zeng M, Gandra S, Mathur S *et al.* Classifying antibiotics in the WHO Essential Medicines List for optimal use-be AWaRe. *Lancet Infect Dis* 2018; **18**: 18–20.

5. Rawson TM, Gowers SAN, Freeman DME, Wilson RC, Sharma S, Gilchrist M *et al.* Microneedle biosensors for real-time , minimally invasive drug monitoring of phenoxymethylpenicillin : a first-in-human evaluation in healthy volunteers. *Lancet Digit Heal* 2019. 7(1);E335-E343

Appendix 1. Summary of study

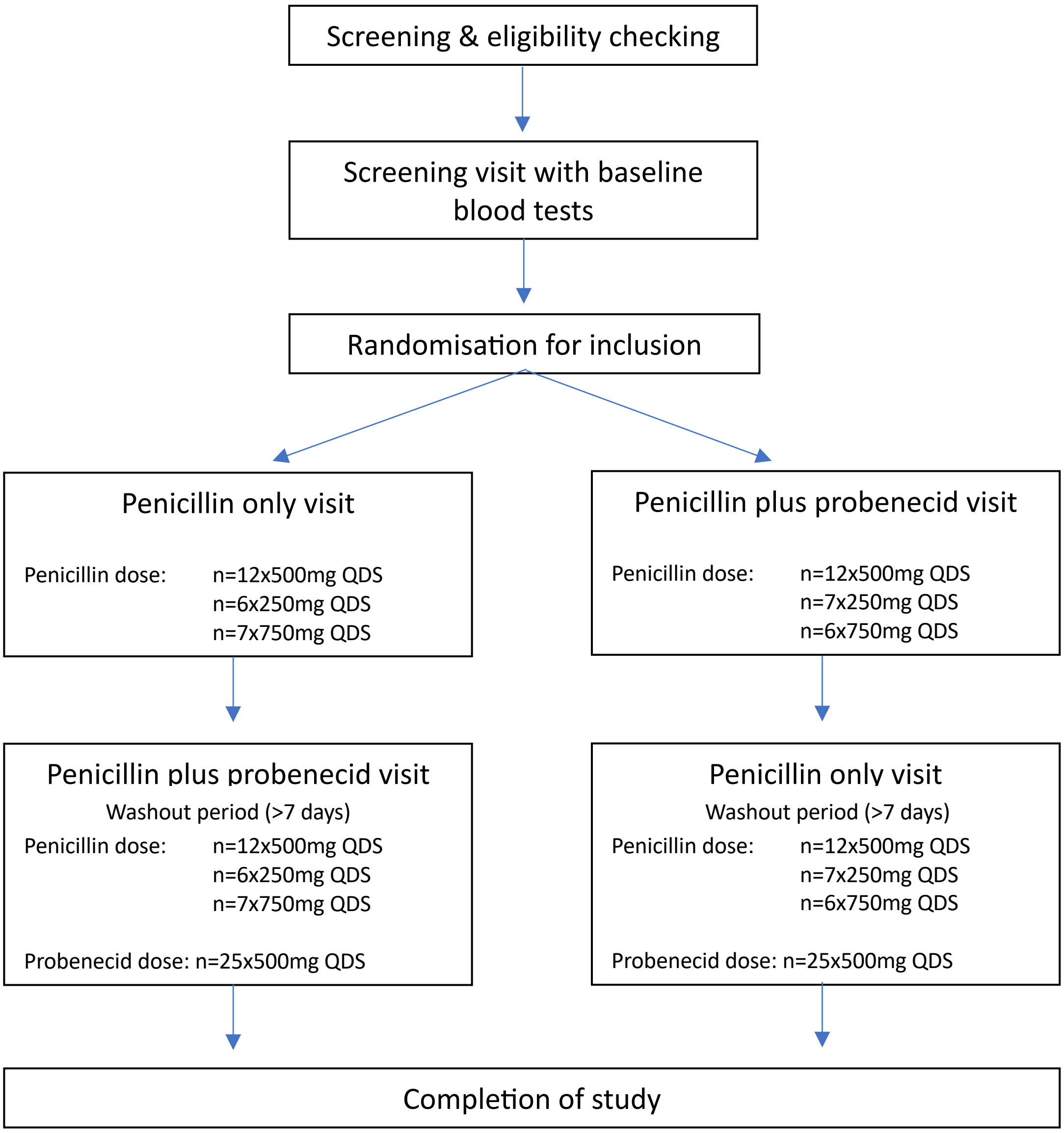

Appendix 2. Summary of investigations, treatments and assessments

	Screening visit	Study visit 1	Study visit 2
**Screening questions**	X		
**Consent obtained**	X		
**History and examination**	X		
**Screening bloods**	X		
**Pregnancy test (if female)**	X		
**Penicillin prescription**		X	
**Penicillin + probenecid prescription**			X
**Diary card for dosing / side effects**		X	X
**Phlebotomy**		X	X
**Documentation and reporting of adverse events**		X	X
**Documentation and reporting of serious adverse events**		X	X
